# Body Mass Index, Haemoglobin, and Total Lymphocyte Count as a Surrogate for CD4 Count in Resource Limited Settings

**DOI:** 10.1155/2017/7907352

**Published:** 2017-04-18

**Authors:** Louis Boafo Kwantwi, Bismark Kwame Tunu, Daniel Boateng, Dan Yedu Quansah

**Affiliations:** ^1^Department of Molecular Medicine, Kwame Nkrumah University of Science and Technology, Kumasi, Ghana; ^2^School of Public Health, Kwame Nkrumah University of Science and Technology, Kumasi, Ghana; ^3^Department of Biomedical and Forensic Sciences, University of Cape Coast, Cape Coast, Ghana

## Abstract

*Background*. In view of the lack of evidence on the possibility of an economically viable, easy, and readily available biomarker to substitute the traditional role of CD4 counts in HIV disease progression, this study seeks to investigate the potential use of body mass index (BMI), haemoglobin (Hb), and total lymphocyte count (TLC) as surrogate biomarkers for monitoring the disease.* Methods*. This cross-sectional study was undertaken at the antiretroviral clinic (ART) of the Bomso Hospital, Kumasi, Ghana. We recruited 384 individuals who were 18 years or older and confirmed HIV seropositive patients. Blood samples were assayed for TLC and Hb. Weight and height were determined and BMI was calculated.* Result*. At a cut-off point of 12.15 g/dL, Hb had sensitivity and specificity of 73.9% and 56.8%, respectively, whereas BMI had 69.6% and 80.1% sensitivity and specificity, respectively. The sensitivity and specificity were also 100% among the studied participants at a cut-off point of 1200 mm^−3^ for TLC. There was a significant positive correlation between CD4 count and Hb (rho 0.262, *p* = 0.0001), BMI (rho 0.301, *p* = 0.0001), and TLC (rho 0.834, *p* = 0.0001).* Conclusion*. The study demonstrates that TLC, Hb, and BMI may provide some useful prognostic information independent of that provided by CD4 count.

## 1. Introduction

HIV/AIDS infection continues to pose threats to patients due its devastating effect on the human CD4 cells [1]. The HIV/AIDS virus's ability to weaken the immune system results in a number of disorders including wasting syndrome [2], hematological derangements such as anemia [3], lymphopenia [4], and thrombocytopenia [5] which are the most common cause of HIV related mortality and morbidity [6].

In recent times one of the greatest challenges in the management and care of HIV in poor resource settings has been the evaluation of CD4 count measurement; a parameter which is seen as a traditional biomarker for predicting the progression and monitoring treatment response to highly active antiretroviral therapy (HAART) [7]. The frequency with which costly CD4 counts are administered to monitor treatment efficacy as well as therapeutic regimens continues to be a burden to many underdeveloped countries; therefore the need for an alternate cost effective, easily performed, and readily available surrogate markers that can assist in predicting the disease progression and patient's response to HAART is needed in these settings.

Among HIV infected individuals, studies have indicated a positive association between higher BMI [8], TLC (1200 cell/ul) [9], and Hb [10] (less than 10 g/dL) with CD4 counts (<350 and <200 cells/mm^3^). A study reports that, being obese or overweight was independently associated with higher CD4 counts, compared to their nonobese (18.5–24.9 kg/m^2^) controls [11]. Decrease in Hb levels correlates positively with CD4 cell counts [12]. Anemia, an independent predictor of HIV/AIDS progression, has been found to improve with antiretroviral treatment [10]. Several studies have also indicated adequate sensitivity and specificity to consider TLC as a surrogate measure for CD4 count [13]. Despite these successes, few studies have evaluated the predictability of BMI, Hb, and TLC as surrogate markers to CD4 counts in Ghana.

In view of the lack of evidence on the possibility of an economically viable, easy, and readily available biomarker to substitute the traditional role of CD4 counts in HIV disease progression, this study investigated the potential use of BMI, Hb, and TLC as surrogate biomarkers for monitoring HIV disease progression in Ghana.

## 2. Methods

### 2.1. Participants and Study Design

This cross-sectional study was undertaken at the ART clinic of the Bomso hospital in the Ashanti region of Ghana from August 2015 to March 2016. We recruited a total of 384 individuals who were 18 years or older and were confirmed HIV seropositive patients at the facility. Our subjects included 208 HIV HAART and 176 HIV HAART naïve patients. Participants who were pregnant and had HIV confections including other opportunistic infections were excluded from the study.

We categorized participants into three groups based on their CD4 lymphocyte counts in accordance with the Center for Disease Control classification. The groups were CD4 counts less than 200 mm^−3^, between 200 and 499 mm^−3^, whereas the third group consisted of patients with CD4 count above 500 mm^−3^. All study protocol was reviewed and approved by the Committee on Human Research and Publication of the Kwame Nkrumah University of Science and Technology (KNUST) and informed consent was also sought from all the participants.

### 2.2. Measurement of BMI

We measured the weight of participants at baseline during their periodic ART clinic visits. The height of subjects was however measured during their first visit only. Height was measured with a stadiometer and rounded to the nearest 0.1 cm. The Tanita HD-351 Scale was used for weight measurement and rounded to the nearest 0.05 kg. BMI was calculated using the formula (body weight in kilograms)/(height in meters)^2^.

### 2.3. Measurement of CD4 Counts, Haemoglobin, and T Lymphocyte

CD4 counts, Hb, and TLC were measured by drawing 3 mL of venous blood from each participant under sterile conditions after applying a tourniquet for less than a minute into anticoagulated sequestrene bottles (EDTA). A complete blood count, including Hb, white blood counts, and lymphocytes with automated differential, was measured. The TLC was calculated by multiplying the white blood count by the automated percent lymphocytes. The full blood count was determined using an autoanalyzer (Cell DNY 1800 from Abbott Diagnostics Division, USA). CD4 analysis was done with the use of the Becton Dickenson and company haematological analyzer called the BD FACSCount from California in USA. The BD FACSCount system used flow cytometry for the quantification of the CD4 T lymphocytes. The laboratory analysis followed a standard flow cytometry performed in laboratories certified by the National Institute of Allergies and Infectious Diseases (NIAID) Flow Cytometry Quality Assessment Program.

### 2.4. Data Analysis

All statistical analyses were performed with SPSS version 22. Data were presented as median interquartile range (IQR) for nonparametric variables whiles grouped variables were expressed as proportions. We performed a Mann-Whitney *U* test to compare the differences between HAART naïve and HAART patients for Hb, BMI, and TLC. Kruskal-Wallis test was performed to determine the extent to which Hb, BMI, and TLC influence HIV disease progression. We further performed a linear regression test to determine the associations that exist between CD4 count and Hb, BMI, and TLC. Suitable cut-off point was determined for Hb, BMI, and TLC using Youden's index. The performance of Hb, BMI, and TLC was assessed using the area under the curve from the receiver operator characteristics. Positive and negative predictive values were also calculated. All statistical significance were accepted at *p* < 0.05.

## 3. Results

### 3.1. Baseline Characteristics of Study Participants

Table 1 shows the demographic and clinical characteristics of the study participants. Out of the three hundred and eighty-four (384) participants, the majority (69.8%) were females in both the HAART and the HAART untreated patients. The median age of the HAART group (41 yrs) was not statistically different from the HAART untreated group (40 yrs). The median CD4 counts of the HAART group (458 mm^−3^) were significantly higher than the HAART untreated group (229 mm^−3^); *p* < 0.0001. The median BMI, Hb, and TLC of HAART patients were significantly different from the HAART untreated patients as detailed in Table 1.

### 3.2. Hb, BMI, and Total Lymphocyte Count in HIV Disease Progression

Table 2 describes the trend of Hb, BMI, and T lymphocyte in the disease progression. Hb, BMI, and TLC were found to decrease significantly (*p* ≤ 0.0001) as the disease progresses with lowest median values among patients with CD4 less than 200 mm^−3^ for Hb (11.4 g/dL), BMI (19.2 kg/m^2^), and TLC (1989 mm^−3^).

### 3.3. Predictive Performance of Haemoglobin, BMI, and Total Lymphocyte Count

Table 3 describes the predictive performance of Hb, BMI, and TLC in predicting HIV disease progression among the studied participants: HAART and the HAART untreated. At a cut-off point of 12.15 g/L, Hb had sensitivity and specificity of 73.9% and 56.8%, respectively, while BMI had 69.6% and 80.1% for sensitivity and specificity, respectively. The sensitivity and specificity were also 100% among the studied participants at a cut-off point of 1200 mm^−3^ for TLC. The area under the curve for Hb, BMI, and TLC was 0.688, 0.780, and 1.000, respectively, for the total study participants. The area under the curve of all the biomarkers was very high in both HAART and HAART untreated patients. In the HAART patients, the area under the curve was 0.679, 0.789, and 1.00 for Hb, BMI, and TLC, respectively, whereas, in the HAART untreated patients, this was 0.658, 0.817, and 1.00 for Hb, BMI, and TLC, respectively.

TLC had the highest positive (91.3%) and negative predictive (87.6%) values followed by BMI (PPV 71.7%, NPV 51.4%). Hb had the lowest positive (67.4%) and negative predictive (21.2%) values.

**Figure 1:**
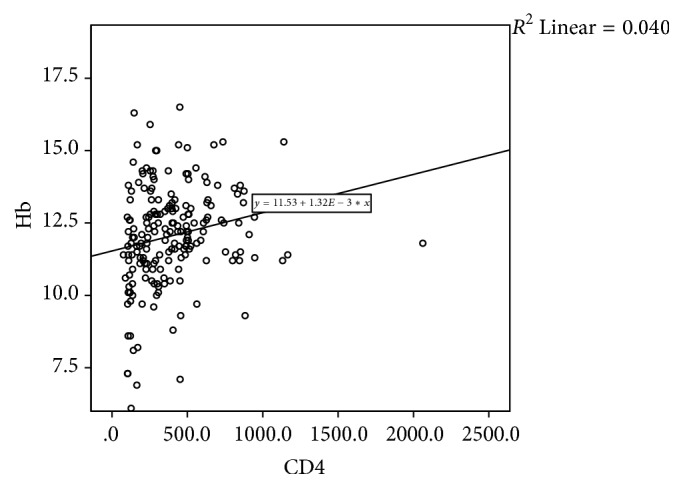
Scatter plots depicting the correlations of CD4 count and haemoglobin (Hb).

**Figure 2:**
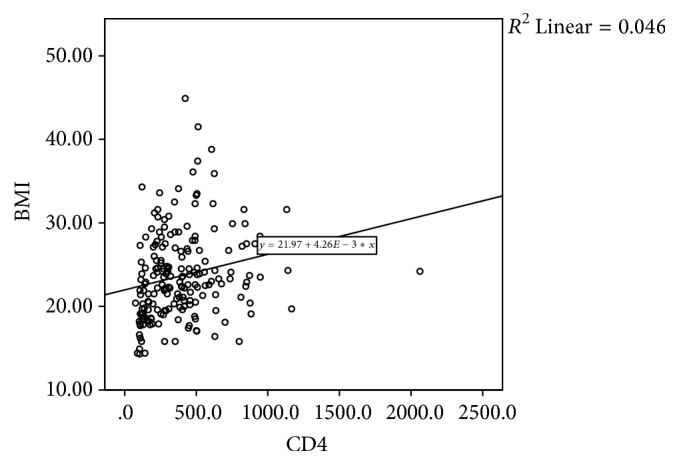
Scatter plots depicting the correlations of CD4 count and body mass index (BMI).

**Figure 3:**
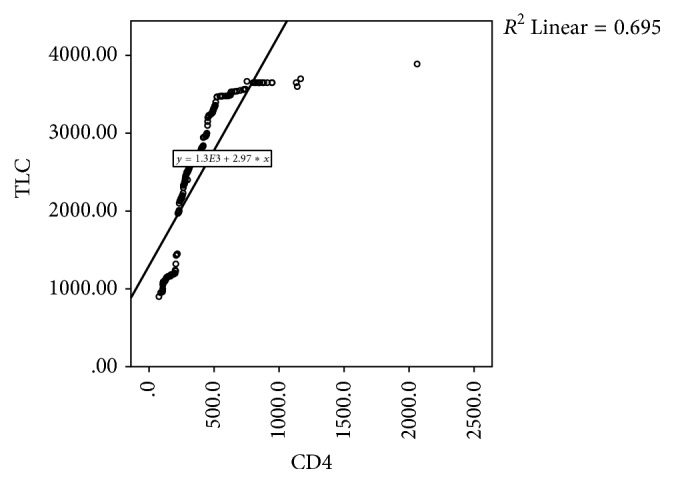
Scatter plots depicting the correlations of CD4 count and total lymphocyte count (TLC).

Figures 1, 2, and 3 are scatter plots depicting the correlations of CD4 count with Hb, BMI, and TLC. There was a significant positive correlation between CD4 count and haemoglobin (rho 0.262, *p* = 0.0001), BMI (rho 0.301, *p* = 0.0001), and TLC (rho 0.834, *p* = 0.0001).

## 4. Discussion

This study was conducted to assess whether Hb, BMI, and TLC can be used as a surrogate for CD4 count. The use of CD4 counts to assess disease progression and to guide treatment among HIV positive persons is well established. The cost implication of CD4 testing however has sprouted the need to find a less expensive surrogate maker in limited resource settings. Previous evidence has shown the possibility of using TLC in this regard, although there has been lack of consensus on the cut-off for sensitivity or specificity. There has also been the call to assess other surrogate biomarkers that could be used in place of CD4 counts. This study sought to access the suitability of Hb, BMI, and TLC as surrogates for CD4 counts, using Youden's *J* index to establish the cuff-off where sensitivity and specificity are the highest.

This study was found TLC to be a good predictor of CD4 counts. At the cut-off of 1200 cells/mm^3^, a sensitivity and specificity of 100% were observed for patients who were not on HAART treatment. The overall diagnostic performance using the AUC was equally very high (AUC = 1) in both HAART treated and HAART untreated groups thus indicating the use of TLC as a potential surrogate marker in remote and deprived areas of Ghana where scarcity of laboratory technologies is a good choice. Total lymphocyte count at a cut-off of 1200 cells/mm^3^ is a good substitute for CD4 < 200 cell/mm^3^ in remote and deprived areas of Ghana: thus, all individuals who need medication would be given the needed medication if a total lymphocyte count of 1200 cells/mm^3^ was used, as recommended by the WHO. Also, patients in the advanced stage of the disease could be identified and given the needed attention and treatment if a cut-off of 1200 cells/mm^3^ is used as a surrogate for CD4 count in our settings. This corroborates previous evidence that also found a high sensitivity and specificity for the same cut-off of TLC [9, 14]. However conflicting results have been reported [15, 16].

Bivariate associations showed a high positive correlation between TLC and CD4 counts and the mean level of TLC increased with increased levels of CD4 for both HAART treated and HAART untreated patients. This is also consistent with the study by Wang et al. [14], among HIV patients from four countries that observed a high positive correlation between TLC and CD4 counts before and after initiating HAART. Similar relationship was observed in other studies from limited resource settings [9, 13]. However, previous study by Mbanya et al. [17] reported a weak association between CD4 count and TLC and hence the limited value of TLC in predicting CD4 counts making it unsuitable to be substituted for CD4 counts. These differences in findings could be as a result of different ethnic, racial, epidemiological, and socioeconomic factors [18] among study participants.

This study further showed that BMI and Hb could also provide some useful prognostic information about HIV disease progression. A sensitivity, specificity, and AUC of 73.9%, 56.8%, and 0.688 for Hb and 69.9%, 80.1%, and 0.780 for BMI, respectively, were observed in predicting CD4 counts < 200 mm^−3^. These levels of AUC based on Youden's *J* index show a strong predictive ability of Hb and BMI at cut-offs of 12.15 kg/m^2^ and 20.65 g/dL, respectively. However, when haemoglobin and BMI are used as a substitute for CD4 at these cut-offs some of the patients who require medication will not be given the needed medication due to relatively low sensitivity and specificity. Further, patients in the advanced stage of the disease cannot be clearly identified and given the needed attention and treatment should these cut-off points be used a surrogate for CD4 count in our settings. There was also a significantly positive correlation between these biomarkers and CD4 counts in the studied population and a trend analysis revealed that an increase in Hb, BMI, and TLC is associated with an increase in the level of CD4 counts. Previous studies [19, 20] have reported higher BMI to be associated with an increased CD4 counts, improved immune reconstitution, and improved survival resulting in a slower disease progression. The trend relationship between Hb and CD4 counts has also been confirmed in previous studies. Earlier reports have also demonstrated that low levels of haemoglobin may be associated with AIDS and death in people with HIV [21, 22] indicating the prognostic value of haemoglobin measurement in the disease progression as evidence in this study. The significant positive correlation observed between haemoglobin levels and CD4 count in the studied participants coupled with the decrease in haemoglobin levels as the disease progress is indicative that haemoglobin measurement may independently provide prognostic information provided by CD4 counts in economically disadvantaged settings.

## 5. Conclusion

The study has shown that TLC, Hb, and BMI may provide some useful prognostic information independent of what is provided by the CD4 count. The regular measurement of these parameters in resource limited settings may therefore be useful to clinicians in monitoring patient's response to antiretroviral therapy and predicting the stage of the disease. This could possibly assist clinicians in identifying patients who are at higher risks of disease progression thereby reducing mortality caused by the HIV infection.

## Figures and Tables

**Table 1:** Demographic and clinical characteristics of the studied participants.

Parameter	Total subjects (384)	HAART group (208)	HAART naïve (176)
Age (yrs.)	40 (34–50)	41 (35–53)	40 (31.3–50)
Sex (*n*, %)			
Male	116 (30.2)	62 (16.1)	54 (14.1)
Female	268 (69.8)	146 (38.0)	122 (31.8)
CD4 (mm^−3^)	346.5 (202–503.3)	458.0 (307.5–633.8)	229.0 (136.3–338.8)
Hb (g/dL)	12.1 (10.2–15.1)	13.4 (12.6–14.2)	10.6 (10.4–12.8)
BMI (kg/m^2^)	22.9 (19.7–29.9)	26.3 (24.3–28.9)	23.5 (19.1–24.9)
T lymphocyte (mm^−3^)	2615 (1232–3336)	3225 (2547–3230)	1989 (1145–2605)

HAART highly active antiretroviral therapy.

**Table 2:** Haemoglobin, BMI, and total lymphocyte count in HIV disease progression.

Parameter	CD4 count			*p* value
	<200	200–499	≥500	
Total				
BMI (kg/m^2^)	19.2 (17.9–21.9)	23.9 (21.1–27.3)	24 (22.1–28.6)	<0.0001
Hb(g/dL)	11.4 (10.1–12.225)	12.2 (11.2–13.1)	12.6 (11.7–13.6)	<0.0001
T lymphocyte (mm^−3^)	1114 (1087–1161)	2595 (2307–2837)	3534 (3476–3650)	<0.0001

HAART group				
BMI (kg/m^2^)	11.8 (11.35–12.15)	12.4 (11.55–13.25)	13.5 (11.65–15.55)	0.153
Hb (g/dL)	21.7 (19.5–24.8)	25.6 (20.4–26.1)	27.1 (23.4–29.9)	0.005
T lymphocyte (mm^−3^)	1122 (1072–1172)	2680 (2490–2990)	3550 (3477–3650)	<0.0001

HAART naïve group				
BMI (kg/m^2^)	10.0 (9.73–11.50)	11.70 (10.90–12.90)	12.0 (10.75–13.15)	0.027
Hb (g/dL)	18.2 (19.2–22.9)	22.5 (20.1–24.6)	23.9 (22.6–27.4)	<0.0001
T lymphocyte (mm^−3^)	1122 (1072–1172)	2680 (2490–2990)	3477 (3394–3509)	<0.0001

**Table 3:** Predictive performance of hemoglobin, BMI, and total lymphocyte count in predicting CD4 counts < 200 mm^−3^.

Parameter	Cut-off	Sensitivity	Specificity	PPV	NPV	AUC
Total						
Hb	12.15	73.9%	56.8%	67.4%	21.2%	0.688
BMI	20.65	69.6%	80.1%	71.7%	51.4%	0.780
Total lymphocyte count	1200	100%	100%	91.3%	87.6%	1.000

HAART						
Hb	12.15	70%	63.8%	52.6%	28.7	0.679
BMI	20.35	60%	77.8%	50%	56.4%	0.787
Total lymphocyte count	1219	90%	100%	100%	81.9%	1.000

HAART naïve						
Hb	10.35	41.7%	90.4%	36.1%	73.1	0.658
BMI	22	77.7%	78.8%	72.2%	80.8%	0.817
Total lymphocyte count	1200	100%	100%	91.7%	100%	1.000

## Abbreviations

BMI: Body mass index
HIV: Human immunodeficiency virus
HAART: Highly active antiretroviral therapy
TLC: Total lymphocyte count.
